# Effects of Particulate Matter on Daily Mortality in 13 Japanese Cities

**DOI:** 10.2188/jea.13.314

**Published:** 2007-11-30

**Authors:** Takashi Omori, Go Fujimoto, Isao Yoshimura, Hiroshi Nitta, Masaji Ono

**Affiliations:** 1 Kyoto University School of Public Health.; 2 BANYU Pharmaceutical Co., Ltd.; 3 Tokyo University of Science.; 4 National Institute for Environmental Studies

**Keywords:** mortality, particulate matter, suspended particulate matter, risk

## Abstract

BACKGROUND: In recent years, numerous reports demonstrating the relationship between an increase in the concentration of fine particulate matter (PM_2.5_) and daily mortality have been released in the United States and Europe. There have been few studies that clearly characterize the short-term effects of particulate matter on the mortality in Japan. We conducted data analysis to investigate the short-term effects of suspended particulate matter (SPM) on mortality in Japan.

METHODS: In this study, we used data sets from the 13 largest cities containing data on the daily mortality of residents aged 65 years or older, concentrations of air pollutants including SPM, temperature, and humidity. Risk ratios for mortality resulting from respiratory diseases, cardiovascular diseases, and all causes other than accidents, from 1990 through 1994, were summarized using a generalized additive model (GAM) and a meta-analysis of random effect model.

RESULTS: The risk ratios for an increase of 10 *μ*g/m^3^ in SPM concentrations adjusted for SO_2_, NO_2_, CO, O_x_, temperature, and humidity were 1.0077 for all causes of mortality, 1.0109 for respiratory diseases, and 1.0091 for cardiovascular diseases, and the lower limits of the 95% confidence intervals for the risk ratios were greater than one for all cases. With regards to the effects of time lag, risk ratios were higher for the SPM concentrations on the day when the mortality was recorded, and the preceding day.

CONCLUSIONS: These results suggest a positive relationship between SPM concentrations and daily mortality in Japan.

In recent years, numerous reports demonstrating the relationship between an increase in the concentration of fine particulate matter (PM), typically PM_2.5_, and daily mortality have been released in the United States and Europe.^1^^-^^5^ Many of these support the finding that an increase in fine particulate matter causes an increase in the daily mortality from respiratory diseases, cardiovascular diseases, and all causes other than accidents. However, there have been few studies that clearly describe the short-term effects of PM on mortality in Japan.

The many types of PM found in the atmosphere are divided into several categories according to particle size. PM_10_ is defined as any particle less than 10 *μ*m in diameter with a 50% cut-off, and PM_2.5_ is defined as having a particle diameter of less than 2.5 *μ*m and the same cut-off as PM_10_. In Japan, suspended particulate matter (SPM) is defined under the National Air Quality Standard as any particle with a diameter of less than 10 *μ*m with a 100% cut-off.^6^ The theoretical 50% cut-off diameter for SPM is assumed to be approximately 7 *μ*m.^7^ The particle diameters of SPM measured in Japan are intermediate to those included in the PM_2.5_ and PM_10_ parameters. Although the daily fluctuations of SPM are similar to those of PM_2.5_,^8^ the constituents of PM could differ among countries.

In Japan, the concentration of sulphur dioxide (SO_2_) and carbon monoxide (CO) has fallen within acceptable limits in recent years. However, SPM, nitrogen dioxide (NO_2_) and oxidant (Ox) has not declined because of the continued increase in automobile use. SPM concentrations were above the National Air Quality Standard at the most of the monitoring stations in large cities.^6^ Furthermore, some analyses^9^ provide little or no support for a threshold for PM mortality effects, and the PM-mortality relationships seem to be observed at low PM concentrations, even those below the air quality standard. Therefore, it is important to investigate the PM-mortality relationships in Japan even though many studies have reported the relationships in the United States and Europe.

In this paper, we provide a quantitative summary of the short-term effects of SPM on mortality using a regression model.

## METHODS

The 13 cities used in this study are designated as major cities by the government from aspect of the population. The states of the air pollution of these cities differ each other, but they are thought to be representative of the cities in Japan in different regions and different levels of air pollution.

We used mortality data obtained from the vital statistics of the Ministry of Health, Labour and Welfare, air pollution data obtained from the National Institute for Environmental Studies, and meteorologic data obtained from Japan Meteorologic Agency. Since elderly people are more sensitive to air pollution, the analyses focused on mortality data collected between 1990 and 1994 for residents aged 65 years or older who had lived in one of the 13 cities observed. Registered address while alive and not place of death was used in the analysis. Note that we refer to the 23 wards of Tokyo as Tokyo here. In 1990, the population of the 13 cities was approximately 27 million, of which 3 million people were 65 years of age or older. Base on the mortality data, deaths resulting from all causes other than accidents (ICD-9: 001-799), deaths resulting from respiratory diseases (480-486, 490-496) and deaths resulting from cardiovascular diseases (390-448) were examined. For our analysis, we used air pollution data collected at monitoring stations located in each city, which were directly managed by the Ministry of the Environment. The air pollution data consisted of the daily means of SPM, SO_2_, NO_2_, CO and O_x_ concentrations. We used meteorologic data collected at monitoring stations located in each city, which were managed by Japan Meteorologic Agency. The meteorologic data consisted of the daily average temperature and relative humidity. The relative humidity data of Fukuoka was used for Kitakyushu because there are no available records of the relative humidity for Kitakyushu.

We developed a risk ratio of the short-term effects of SPM on mortality in Japan by a two-stage approach; first we applied regression analyses to estimate the risk ratios of SPM adjusted for confounding factors for each city, and second we constructed the integrated risk ratio for each cause of death. We employed two types of regression models to estimate the risk ratio: the generalized additive model (GAM)^10^ and the generalized liner model (GLIM). Because the value of the risk ratio is dependent on the regression model used, a more flexible regression model is desirable. We chose the GAM regression model to estimate the risk ratio because GAM has more parameters than GLIM. However, we also calculated the risk ratio based on GLIM to investigate robustness of the result of GAM. Because the risk ratio values for each city were not of interest, each risk ratio should be regarded as a random sample from the population in Japan. Then we estimated the integrated risk ratios using the meta-analysis technique with a random effect model^11^ in the second stage.

In addition, we investigated the effects of the risk ratios using regressors with a time lag since death may be caused by SPM of the previous day rather than of the day the mortality was recorded.

Calculations for GAM and GLIM were performed using the PROC GAM and PROC GENMOD, respectively, of the SAS^®^ statistical software package version 8.2 (SAS Institute Inc. NC, USA).

For each city, we employed GAM and GLIM for daily mortality with the log link function as the regressand and modeled by the concentration of pollutants including SPM, temperature, and relative humidity as the regressors. When using GAM, the effects were estimated as being linear for SPM and non-liner smooth functions for SO_2_, NO_2_, CO and O_x_ concentrations, temperature, and relative humidity. In the case of GLIM, all parameters were estimated as being linear, whereas linear functions consisting of the original scale and square scale are employed for temperature for each city:

$$\ln(\mu )=\beta_{0}+\beta_{\text{SPM}}\;SPM+f_{1}(\text{temp})+f_{2}(\text{humi})\;\text{for GAM},$$ (1)

$$\ln(\mu )=\beta_{0}+\beta_{\text{SPM}}\;SPM+B\;(\text{temp})+\beta_{humi}\text{ humi}\;\text{for GAM},$$ (1')

$$\ln(\mu )=\beta_{0}+\beta_{\text{SPM}}\;SPM+f_{1}(\text{temp})+f_{2}(\text{humi})+f_{3}(\text{SO}{}_{2})+f_{4}(\text{NO}{}_{2})+f_{5}(\text{CO})+f_{6}(\text{O}{}_{x})\;\text{for GAM},$$ (2)

$$\ln(\mu )=\beta_{0}+\beta_{\text{SPM}}\;SPM+B\;(\text{temp})+\beta_{humi}\text{ humi}+\beta_{\text{SO}{}_{2}}\text{SO}{}_{2}+\beta_{\text{NO}{}_{2}}+\beta_{\text{CO}{}_{}}\text{CO}+\beta_{\text{O}{}_{x}}\text{O}{}_{x}\;\text{for GAM},$$ (2')

where *μ* denotes the expected value of daily mortality; SPM, SO_2_, NO_2_, CO, O_x_, temp, and humi represent the concentration of SPM, SO_2_, NO_2_, CO, O_x_, temperature, and relative humidity, respectively; *β*_0_, *β*spm, *β*_SO2_, *β*_NO2_, *β*_CO_, *β*_ox_, and *β*_humi_ represent the linear effects of the intercept, SPM, SO_2_, NO_2_, CO, O_x_, and relative humidity, respectively; f_i_(.) represent the spline functions for each regressor (i=1,…,5), the smoothing parameters for which were selected using the generalized cross validation method; and B(temp) represents the linear effect of temperature or linear functions that are dependent on temperature or the square of temperature, which varies by city.

For the investigation of time lag, we used the following GAM:

$$\ln(\mu )=\beta_{0}+\beta_{\text{SPM}}\;{SPM}_{\text{j}}+f_{1}({\text{temp}}_{\text{j}})+f_{2}({\text{humi}}_{\text{j}}),$$ (3)

where SPM_j_, tempj and humi_j_ denote the j-day lag of SPM, temperature and relative humidity respectively, and other parameters are the same as above.

For the both models, the risk ratios of mortality, exp(10*β_SPM_*), were calculated for SPM concentration increments of 10 *μ*g/m^3^ for each city.

## RESULTS

Table 1 shows the total population statistics and a breakdown of the population aged 65 years or older in 1990, along with the average daily mortality data for all causes, respiratory diseases and cardiovascular diseases in the 65 years or older population between the years 1990 and 1994 for all the 13 cities.

**Table 1.** Statistics of the population and mortality in study area City

City		Population ^1)^		Number of daily mortality ^2)^		
	
		All age	65 years or older	All causes	Respiratory Diseases	Cardiovascular Diseases
1	Sapporo	1,672	152	16.3	2.5	6.2
2	Sendai	918	80	8.4	1.0	3.4
3	Chiba	829	61	6.7	0.9	2.8
4	Tokyo	8,164	911	103.3	15.0	42.0
5	Yokohama	3,220	278	30.4	4.3	12.4
6	Kawasaki	1,174	94	10.0	1.5	3.9
7	Nagoya	2,155	222	25.6	3.0	11.2
8	Kyoto	1,461	185	22.0	3.1	8.3
9	Osaka	2,624	306	36.9	5.4	13.9
10	Kobe	1,477	169	20.1	2.6	7.7
11	Hiroshima	1,086	107	11.7	1.5	4.6
12	Kitakyushu	1,026	130	14.8	2.1	5.9
13	Fukuoka	1,237	113	12.2	2.0	4.6

Total		27,043	2,808	318.4	44.9	126.9

1) thousand; in 1990

2) person; daily mean mortality (65+ years) between 1990 and 1994

**Figure 1.**
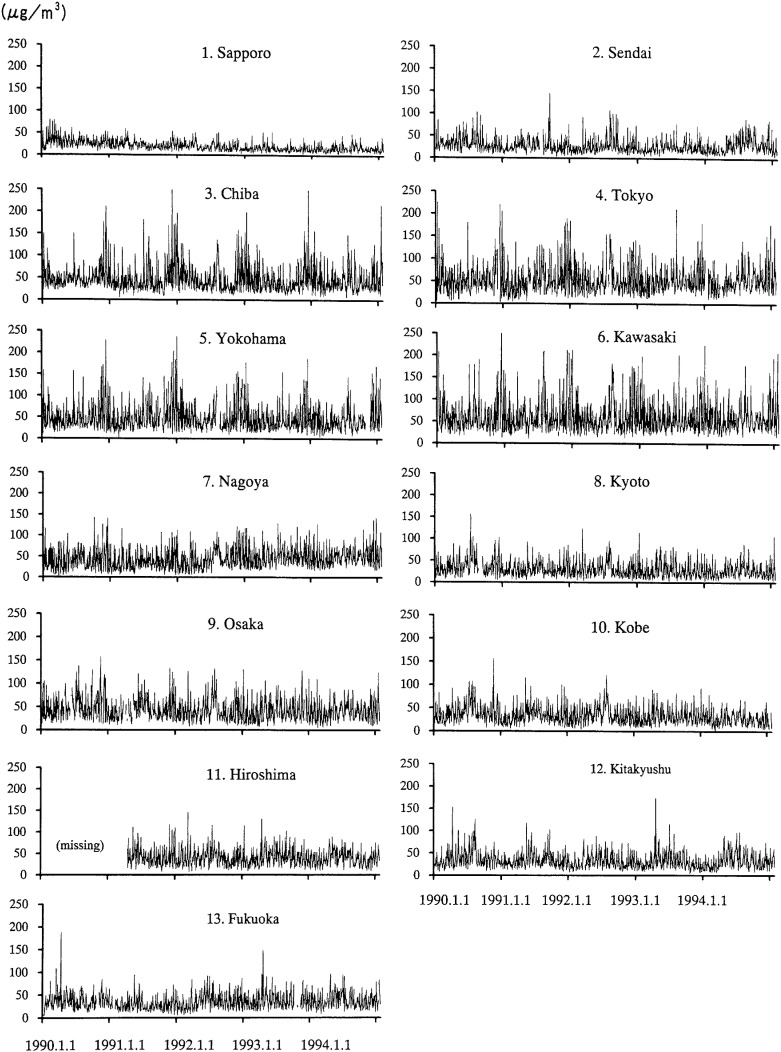
Time trend of the SPM concentrations in 13 cities Each line shows the time trend of daily averages of suspended particulate matter (SPM) concentration in each city where data was collected for the analyses.

Table 2 summarizes the range of the annual mean SPM, SO_2_, NO_x_ CO, and O_x_ concentrations, and the temperature and relative humidity obtained at monitoring stations in each city, and the correlation coefficients between them. Because the correlation coefficients between NO_2_, NO, and NO_x_ are high, we used only NO_2_ in the analysis. Figure 1 shows the time trends of the SPM concentrations for each city.

**Table 2.** Summary statistics of the air pollutants, temperature, and humidity.


	Mean	Correlation							

Sulphur dioxide (SO_2_)	4.9 - 10.5 (ppb)								
Nitrogen oxide (NOx)	24.2 - 110.1 (ppb)	0.45 - 0.81							
Nitrogen monooxide (NO)	7.9 - 62.4 (ppb)	0.27 - 0.75	0.93 - 0.98						
Nitrogen dioxide (NO_2_)	16.4 - 47.7 (ppb)	0.57 - 0.79	0.76 - 0.94	0.59 - 0.84					
Carbon monoxide (CO)	0.5 - 2.5 (ppm)	0.17 - 0.75	0.38 - 0.92	0.34 - 0.89	0.36 - 0.86				
Oxidant (O_x_)	10.6 - 27.0 (ppb)	-0.14 - 0.16	-0.58 - -0.17	-0.56 - -0.25	-0.47 - 0.10	-0.47 - -0.14			
Suspended particular matter (SPM)	20.8 - 59.8 (*μ*g/m^3^)	0.26 - 0.73	0.32 - 0.74	0.20 - 0.68	0.40 - 0.77	0.27 - 0.85	-0.17 - 0.05		
Temperature	9.4 - 17.1 (°C)	-0.58 - 0.17	-0.48 - -0.05	-0.41 - -0.13	-0.55 - 0.04	-0.34 - 0.26	-0.66 - 0.34	-0.08 - 0.42	
Relative humidity	62.6 - 70.7 (%)	-0.42 - 0.04	0.23 - 0.36	-0.17 - 0.35	-0.29 - 0.30	-0.06 - 0.44	-0.54 - -0.15	-0.01 - 0.30	0.05 - 0.49

		SO_2_	NO_x_	NO	NO_2_	CO	O_x_	SPM	Temperature

Table 3 shows the risk ratios for SPM and the 95% confidence intervals (CIs) with regard to the mortality from all causes, respiratory diseases, and cardiovascular diseases. The risk ratio adjusted for temperature and humidity of 1.0049 (95% IC: 1.0038-1.0060) was obtained for all causes, 1.0079 (95% CI: 1.0051-1.0108) for respiratory diseases, and 1.0054 (95% CI: 1.0037-1.0071) for cardiovascular diseases.

**Table 3.** Risk ratio and 95% confidence interval of mortality

		SPM ^1)^		SPM + other pollutants ^2)^	
	
		RR ^3)^	95% confidence interval	RR ^3)^	95% confidence interval
All causes	Integrated	1.0049	1.0038 - 1.0060	1.0077	1.0056 - 1.0099
	Sapporo	0.9821	0.9714 - 0.9930	0.9731	0.9476 - 0.9993
	Sendai	1.0069	0.9957 - 1.0183	1.0218	1.0057 - 1.0381
	Chiba	1.0041	0.9982 - 1.0100	0.9887	0.9778 - 0.9997
	Tokyo	1.0057	1.0041 - 1.0073	1.0088	1.0053 - 1.0124
	Yokohama	1.0024	0.9995 - 1.0054	1.0081	1.0024 - 1.0138
	Kawasaki	1.0058	1.0016 - 1.0101	1.0101	1.0012 - 1.0190
	Nagoya	1.0075	1.0033 - 1.0117	1.0148	1.0071 - 1.0225
	Kyoto	1.0025	0.9969 - 1.0082	0.9969	0.9873 - 1.0066
	Osaka	1.0054	1.0018 - 1.0090	1.0075	0.9997 - 1.0155
	Kobe	1.0035	0.9976 - 1.0094	1.0090	0.9990 - 1.0192
	Hiroshima	1.0113	1.0026 - 1.0201	1.0114	0.9977 - 1.0253
	Kitqkyushu	1.0038	0.9969 - 1.0108	1.0028	0.9941 - 1.0115
	Fukuoka	1.0055	0.9978 - 1.0132	1.0134	1.0025 - 1.0245

	GLIM	1.0051	1.0039 - 1.0063	1.0073	1.0049 - 1.0097

“Respiratory Diseases”	Integrated	1.0079	1.0051 - 1.0108	1.0109	1.0047 - 1.0170
	Sapporo	0.9714	0.9445 - 0.9991	0.9738	0.9104 - 1.0416
	Sendai	1.0331	1.0009 - 1.0662		
	Chiba	0.9979	0.9823 - 1.0138		
	Tokyo	1.0060	1.0018 - 1.0103	1.0122	1.0030 - 1.0216
	Yokohama	1.0076	0.9998 - 1.0154	1.0146	0.9996 - 1.0298
	Kawasaki	0.9917	0.9807 - 1.0028		
	Nagoya	1.0141	1.0019 - 1.0265	1.0400	1.0173 - 1.0633
	Kyoto	0.9986	0.9836 - 1.0137	0.9889	0.9639 - 1.0146
	Osaka	1.0129	1.0035 - 1.0223	1.0012	0.9807 - 1.0222
	Kobe	1.0061	0.9896 - 1.0228	0.9912	0.9638 - 1.0193
	Hiroshima	1.0601	1.0362 - 1.0846		
	Kitqkyushu	1.0476	1.0287 - 1.0667	0.9906	0.9678 - 1.0140
	Fukuoka	1.0233	1.0043 - 1.0425	1.0364	1.0092 - 1.0643

	GLIM	1.0074	1.0043 - 1.0105	1.0114	1.0052 - 1.0176

“Cardiovascular Diseases”	Integrated	1.0054	1.0037 - 1.0071	1.0091	1.0057 - 1.0125
	Sapporo	0.9808	0.9634 - 0.9985	0.9787	0.9372 - 1.0220
	Sendai	0.9979	0.9801 - 1.0160	1.0013	0.9762 - 1.0271
	Chiba	1.0029	0.9937 - 1.0121	0.9928	0.9756 - 1.0102
	Tokyo	1.0062	1.0037 - 1.0087	1.0111	1.0055 - 1.0166
	Yokohama	1.0016	0.9969 - 1.0062	1.0071	0.9982 - 1.0160
	Kawasaki	1.0084	1.0016 - 1.0152	1.0149	1.0005 - 1.0294
	Nagoya	1.0041	0.9978 - 1.0104	1.0091	0.9976 - 1.0207
	Kyoto	1.0060	0.9968 - 1.0152	0.9982	0.9824 - 1.0142
	Osaka	1.0067	1.0009 - 1.0126	1.0173	1.0044 - 1.0305
	Kobe	1.0156	1.0060 - 1.0253	1.0230	1.0065 - 1.0398
	Hiroshima	1.0130	0.9989 - 1.0273	1.0087	0.9865 - 1.0314
	Kitqkyushu	1.0076	0.9967 - 1.0187	1.0127	0.9989 - 1.0267
	Fukuoka	0.9966	0.9840 - 1.0094	0.9889	0.9710 - 1.0070

	GLIM	1.0055	1.0037 - 1.0073	1.0081	1.0045 - 1.0118

1) Risk ratios of suspended particulate matter (SPM) adjusted for temperature and humidity

2) Risk ratios of SPM adjusted for SO_2_, NO_2_, CO, O_x_, temperature, and humidity

3) Risk ratios with an incease in SPM of 10 *μ*g/m^3^

GLIM: generalized liner model

Table 3 also shows the risk ratios generated using GAM in each of 13 cities before integration in the second stage. The risk ratios for all causes for the 12 cities excluding Sapporo were greater than one. The maximum value of the risk ratio was 1.0113 (95% CI: 1.0026-1.0201) for Hiroshima, and the minimum value was 0.9821 (95% CI: 0.9714-0.9930) for Sapporo. For respiratory diseases, risk ratios of Sapporo, Chiba, Kawasaki and Kyoto were less than one. For cardiovascular diseases, risk ratios of all cities excluding Sapporo, Sendai and Fukuoka were greater than one.

As shown in Table 3, the risk ratio adjusted for SO_2_, NO_2_, CO, O_x_, temperature, and humidity of 1.0077 (95% CI: 1.0056-1.0099) was obtained for all causes, 1.0109 (95% CI: 1.0047-1.0170) for respiratory diseases, and 1.0091 (95% CI: 1.0057-1.0125) for cardiovascular diseases.

Table 3 also shows the risk ratios generated using GAM in each of 13 cities before integration in the second stage. The risk ratios from all causes for all cities excluding Sapporo, Chiba and Kyoto were greater than one. The maximum value of the risk ratio was 1.0218 (95% CI: 1.0057-1.0381) for Sendai, and the minimum value was 0.9731 (95% CI: 0.9476-0.9993) for Sapporo.

For respiratory diseases, the risk ratios of Sendai, Chiba, Kawasaki and Hiroshima were not obtained because the algorithm used to estimate parameters on GAM failed to converge. Risk ratios for Sapporo, Kyoto, Kobe and Kitakyushu were all less than one. For cardiovascular diseases, the risk ratios for all cities excluding Sapporo, Chiba, Kyoto and Fukuoka were greater than one. While the CIs for respiratory diseases were wider than for all causes, the pattern for the risk ratios was similar to those for mortality from all causes with the exception of Fukuoka.

**Figure 2.**
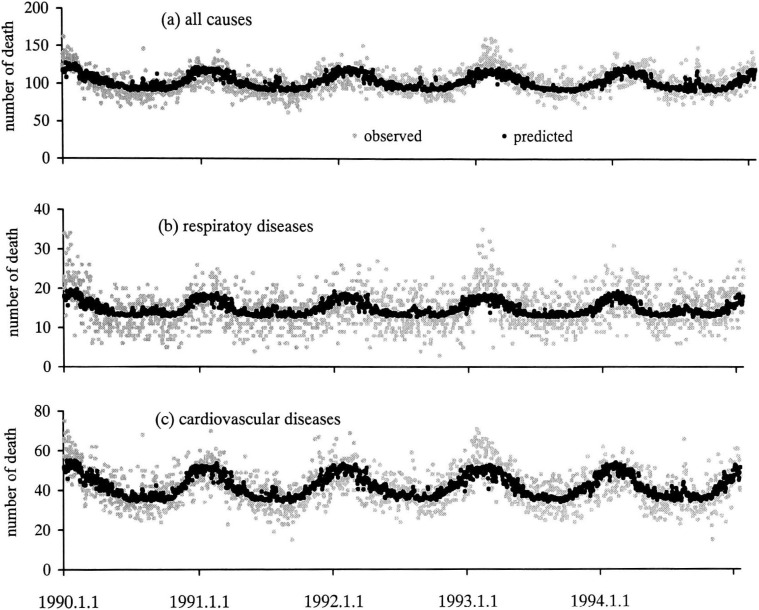
Observed and predicted value of daily mortality against time (in days) in Tokyo In this figure, the daily observed (circle) and predicted (dot) number of mortalities are plotted against time (in days).

Figure 2 shows the observed and predicted values of daily mortality against time (in day) in Tokyo for each mortality category. The predicted values from our model match well with the average trends of data.

In order to investigate the robustness of our analysis, we estimated risk ratios using GLIM place of GAM. The risk ratios and 95% CIs of SPM adjusted for temperature and humidity for mortality from all causes, respiratory diseases and cardiovascular diseases were 1.0051 (95% CI: 1.0039-1.0063), 1.0074 (95% CI: 1.0043-1.0105), and 1.0055 (95% CI: 1.0037-1.0073), respectively. With adjustments of SPM for SO_2_, NO_2_, CO, O_x_, temperature, and humidity, the risk ratios were 1.0073 (95% CI: 1.0049-1.0097), 1.0114 (95% CI: 1.0052-1.0176), and 1.0081 (95% CI: 1.0045-1.0118), respectively (Table 3). The risk ratios values from both GAM and GLIM were very similar.

In order to investigate that the possibility that the effects of SPM concentration on mortality are not caused by the SPM concentration on the day the mortality was recorded, but from SPM concentrations on preceding days, we investigated the integrated risk ratio of SPM for each of the preceding 5 days.

**Figure 3.**
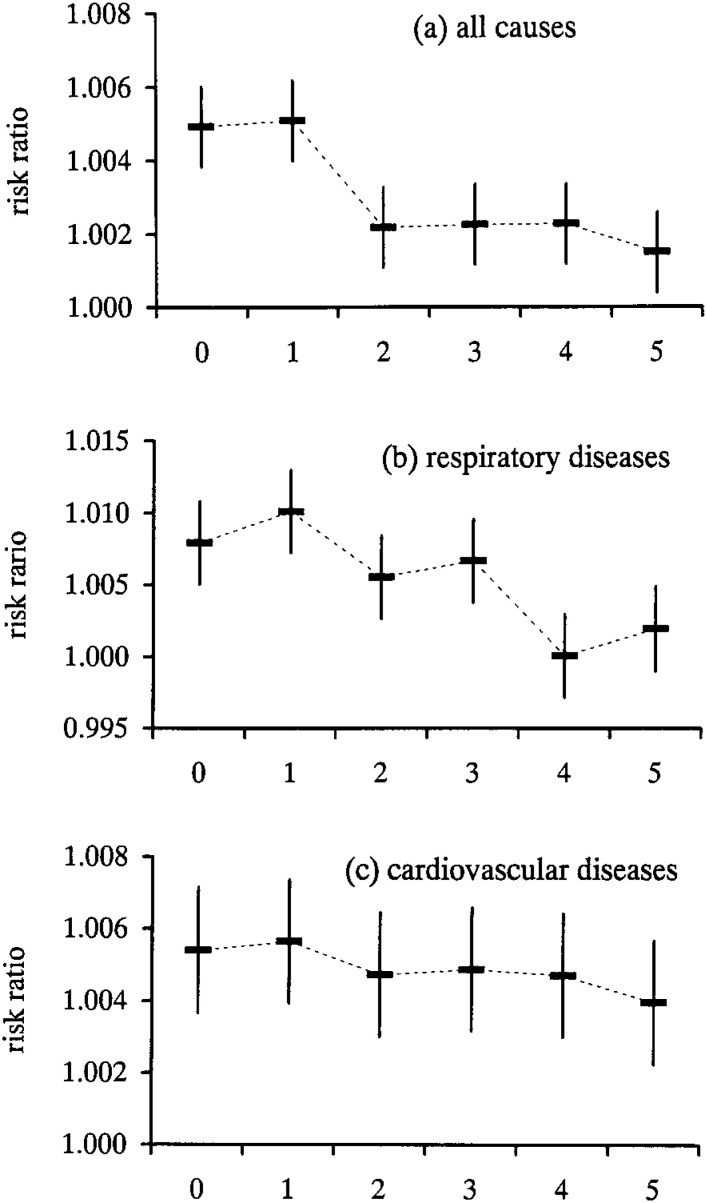
Risk ratios and 95% confidence intervals with the time lag effects The risk ratios are shown as the increase of mortality for an increase in suspended particulate matter (SPM) of 10 mg/m3. The risk ratios were higher for the SPM concentrations on the same day and the previous day.

Figure 3 demonstrate that for all causes of mortality, the risk ratios for the SPM were higher on the same day and on the previous day.

## DISCUSSION

We used air pollution data from monitoring stations in each city. Using the data at the monitoring stations as a surrogate of personal exposure is widely accepted in epidemiologic studies like this one. There was good correlation among the concentrations of each pollution parameters at different monitoring stations within each city used in this analysis.

Earlier studies of air pollution episodes have indicated that high levels of ambient air pollutants can cause deaths. One of the most severe episodes, the London Big Smoke of 1952, is estimated to have caused thousands of deaths primarily in children and in the elderly with lung or heart diseases.^12^ Thereafter, most researchers of air pollution and mortality have focused on these susceptible persons, especially the elderly, in investigating the relationships between air pollution and mortality associated with respiratory and/or cardiovascular disease. We also followed this approach and used respiratory and cardiovascular mortality as well as total non-accidental mortality for the elderly aged 65 years and over.

Because the risk ratios are calculated using regression models, it is important to check the fit. As shown in Figure 2 the predicted values from our model match well with the average trends of data.

For Sapporo, risk ratios and the upper limits of 95% CI for all mortality categories were smaller than one. Though we first suspected a poor fit of the model to the data in the first stage in our estimation, the value of deviance (the statistical index that measures the degree of fit of the model to the data) for Sapporo data were not extremely large compared to those of other cities. Sapporo is the northernmost area in our investigation and has a much lower annual average temperature than other areas. Katsouyanni et al.^13^ reported that the effect size estimates were larger in warmer countries, but other studies have found little difference. The effect of temperature on risk ratios remains inconclusive. Further, the annual average SPM concentrations in Sapporo are lower than the other areas, and the range of SPM concentrations is much narrower than other areas (Figure 1). The range of the regressor affects the estimator in regression analysis. Therefore, we consider the phenomenon is due to lower and narrow range of SPM values in Sapporo compared with the other areas.

For respiratory diseases, some risk ratios could not be estimated by GAM in the first step of our estimation because the algorithm for estimating the parameters failed to convergence, whereas risk ratios could be estimated using GLIM for all cities. Using GAM demands many parameters in exchange for few restrictions on statistical model compared with GLIM, and therefore estimates could not be made for cities such as Sendai, Chiba, Kawasaki and Hiroshima with low daily mortality values (Table 1). However, as shown in the Results, almost identical results were obtained when using both GAM and GLIM. Therefore, we consider that the risk ratio summarized from the calculable risk ratios by GAM represents the risk ratio of urban areas in Japan.

PM is one of the constituents along with other gaseous pollutants in ambient air. It is important to consider the extent to which ambient PM affects health independent of the gaseous co-pollutants. In comparing the risk ratios for the SPM adjusted for temperature and humidity and the risk ratios adjusted for SO_2_, NO_2_, CO, O_x_, temperature, and humidity, it was found that the latter tended to acquire large values and wide 95% CIs. These trends were acknowledged in both models (GAM and GLIM). These findings suggest that observed health effects can be independently attributed to SPM. However, the interactions between PM and gaseous pollutants in both ambient air and in the human body are very complex. Previous findings do not show consistent risk estimates for PM and co-pollutants.^14^

With respect to the effect of time lags, the effects of pollutant concentrations on the previous day (lag 1) were observed in addition to those on the same day (lag 0). Domonici et al.^15^ reported that for the overall pooled results of PM_10_ effects with lags of 0, 1, and 2 days in 90 cities in the United States, the largest lag effect was observed at 1 day. However, the PM_10_ effects in individual cities sometimes showed the largest lag effect at lags 0 or 2. Several studies showed differences in the lag structure between PM and cause-specific mortality. For respiratory deaths, the PM concentrations on the preceding days (i.e., lag 1, 2, or more) had a larger effect than the same-day concentrations. For cardiovascular deaths, immediate responses appear to contribute to the deaths. However, we could not clarify the lag effect for cause-specific mortality from our analyses. The concentrations of SPM and other pollutants on preceding days produced stronger correlations than for concentrations on the same day, and the day-to-day pattern of air pollution may also vary by pollutant. We are planning further investigations of modeling by species and analyses of the lag structure.

Our results were similar to those reported in the comprehensive Samet et al.^14^ investigation of the relationship between daily mortality and PM_10_ data for 90 cities in the United States. For example, the risk ratio for mortality from all causes with an increase in PM_10_ of 10 *μ*g/m^3^ calculated using their results is approximately 1.0041 (note: this value was recently corrected to 1.0027^16^). Another study conducted by Klemm and Mason^17^ demonstrated 1.012 as the risk ratio for mortality from all causes with an increase in PM_2.5_ of 10 *μ*g/m^3^. If we consider the difference in the particle diameter between PM_10_, PM_2.5_ and SPM (approximately PM_7_), the magnitude of the risk of death obtained in our study (1.0049) is almost identical to or intermediate of these studies.

In conclusion, this study is the first investigation of the short-term effect of the concentrations of particulate matter on daily mortality conducted in Japan. The study was performed by similar methods as used in studies conducted recently overseas. The estimated risk ratios for mortality from all causes, respiratory diseases, and cardiovascular diseases were found to be greater than one, with the lower limits of the 95% CI also being greater than one. Our results suggest a positive relationship between SPM concentration and daily mortality in Japan.
